# Characterizing the temporal evolution of altered cardiac mechanics in diet-induced obese mice using cine DENSE CMR

**DOI:** 10.1186/1532-429X-16-S1-P297

**Published:** 2014-01-16

**Authors:** Christopher M Haggerty, Andrea C Mattingly, Cassi M Binkley, Sage P Kramer, David Powell, Frederick H Epstein, Brandon K Fornwalt

**Affiliations:** 1 Departments of Pediatrics, Physiology, and Medicine, University of Kentucky, Lexington, Kentucky, USA; 2 Department of Biomedical Engineering, University of Kentucky, Lexington, Kentucky, USA; 3 Departments of Biomedical Engineering and Radiology, University of Virginia, Charlottesville, Virginia, USA

## Background

Obesity and metabolic syndrome are associated with increased risk of cardiovascular disease. Research suggests that altered cardiac mechanics (i.e., reduced strains, torsion, and synchrony of contraction) are present in obesity; yet, the causes of this mechanical dysfunction and its relationship to other sequelae of obesity (e.g., hypertension and elevated blood glucose) are not well understood. We hypothesize that diet-induced obesity in mice leads to reductions in measures of left ventricular (LV) mechanics, which develop in acute response to the onset of hyperglycemia, hypertension, and ventricular remodeling.

## Methods

Twenty 4-week-old C57BL/6J mice were randomized (n = 10 per group) to either a high-fat (60% kcal from fat) or sucrose-matched low-fat (10% kcal from fat) diet for 28 weeks. After 4 weeks and every 6 weeks thereafter, LV mechanics were quantified using cine displacement encoding with stimulated echoes (DENSE) on a 7T ClinScan MRI (Bruker, Ettlingen, Germany) with a 4-element phased array cardiac coil. Three short-axis and two long-axis slices were acquired with 13-20 frames per cardiac cycle. Semi-automated post-processing was performed using custom software in MATLAB (Mathworks, Natick, MA). Additionally, systolic blood pressure (via tail cuff measurement) and fasting blood glucose were assessed every 4 weeks on staggered schedules.

## Results

**Figure 1:**
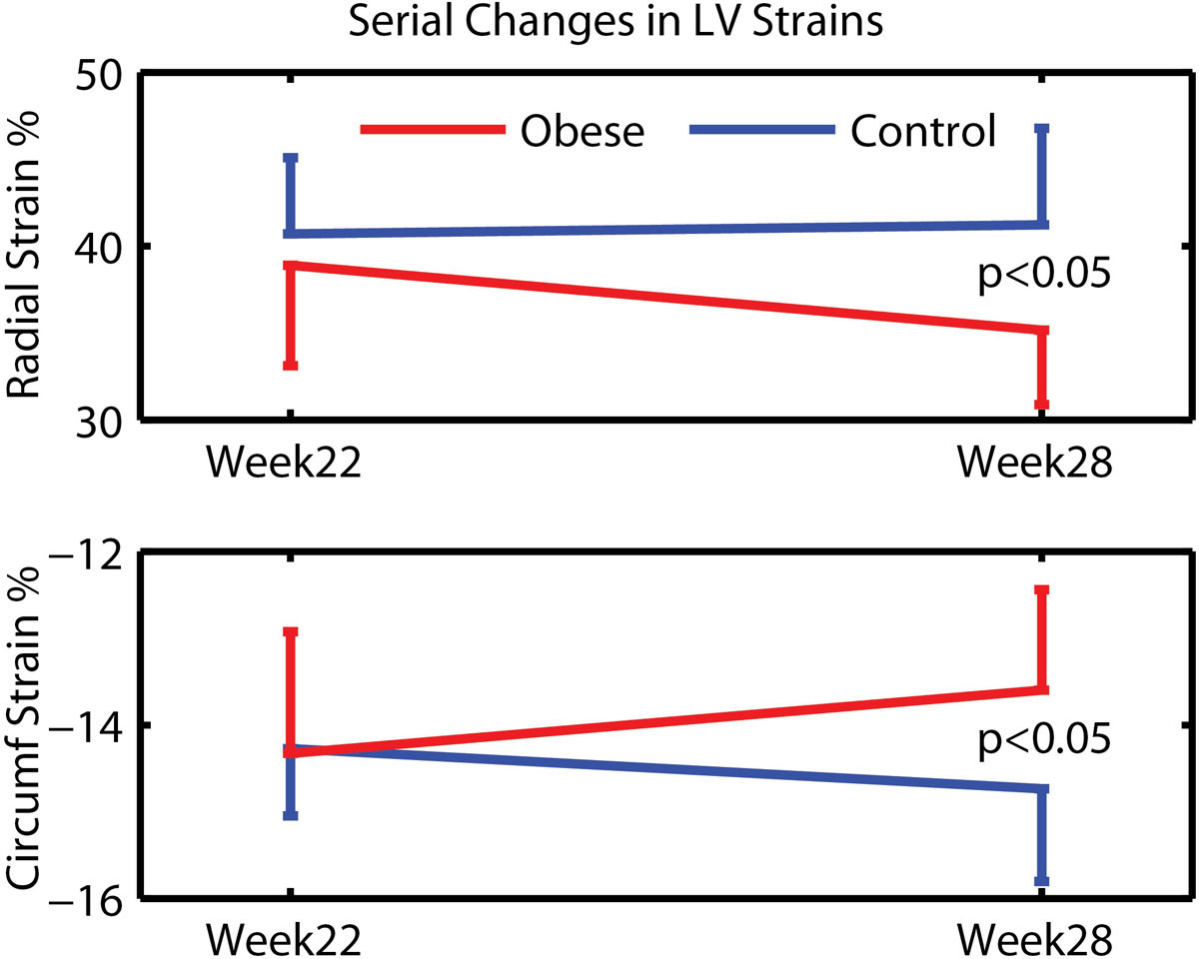


Mice on the high-fat diet became obese relative to the low-fat controls (49.9 vs. 29.2 g, respectively, by week 28; Table 1). Fasting blood glucose was elevated in the high-fat group (202 vs. 112 mg/dL; p < 0.05) starting from the earliest measurement (week 7 on diet), whereas significant differences in LV mass (88 vs. 79 mg) and systolic blood pressure (172 vs. 162 mmHg) developed much later (weeks 22 and 25 on diet, respectively). Significant reductions in peak LV radial (15%) and circumferential (8%) strains (Figure 1) and reduced contractile synchrony were detected in the high-fat group for the first time in week 28. A 10% reduction in peak torsion was also observed at that time, but did not reach statistical significance (p = 0.075). There were no differences in LV cavity volumes or ejection fraction.

**Table 1:** 

Week 28 Measures	Low-fat (n = 10)	High-fat (n = 10)	p-value
Body Mass (g)	29.2 ± 2.0	49.9 ± 7.5	< 0.001

Fasting Blood Glucose (mg/dL)	134.1 ± 17.6	204.1 ± 30.9	< 0.001

Systolic Blood Pressure (mmHg)	161.7 ± 3.4	171.6 ± 8.2	0.0155

LV Mass (mg)	81.6 ± 5.2	95.9 ± 13.0	0.007

End Diastolic Volume (μL)	48.5 ± 7.2	49.2 ± 6.6	0.83

End Systolic Volume (μL)	17.4 ± 3.6	18.1 ± 2.7	0.64

Ejection Fraction (%)	64 ± 4	63 ± 5	0.56

## Conclusions

Diet-induced obesity in mice is associated with reduced left ventricular mechanics. This dysfunction develops long after the manifestation of hyperglycemia in this model, which suggests that chronic alterations in glucose/insulin levels and/or signaling may contribute more to cardiac contractile dysfunction than acute elevations. Late development of concentric ventricular hypertrophy and hypertension prior to suppressed cardiac mechanics also suggests an important role of these processes in the reduced ventricular function.

## Funding

This work was supported by a Postdoctoral Fellowship through the Ruth L. Kirschstein National Research Service Award (5T32HL91812-05), the NIH Director's Early Independence Award (1DP5OD012132-01), a pilot grant from an Institutional Development Award (IDeA) from the National Institute of General Medical Sciences of the NIH (8 P20 GM103527-05), the University of Kentucky Cardiovascular Research Center, grant number UL1RR033173 [TL1 RR033172, KL2 RR033171] from the National Center for Research Resources (NCRR), funded by the Office of the Director, National Institutes of Health (NIH) and supported by the NIH Roadmap for Medical Research, and contributions made by local businesses and individuals through a partnership between Kentucky Children's Hospital and Children's Miracle network.

